# Assessment of Air Contamination by SARS-CoV-2 in Hospital Settings

**DOI:** 10.1001/jamanetworkopen.2020.33232

**Published:** 2020-12-23

**Authors:** Gabriel Birgand, Nathan Peiffer-Smadja, Sandra Fournier, Solen Kerneis, François-Xavier Lescure, Jean-Christophe Lucet

**Affiliations:** 1 National Institute of Health Research Health Protection Research Unit in Healthcare Associated Infection and Antimicrobial Resistance, Imperial College London, London, United Kingdom; 2 Centre Hospitalo-Universitaire de Nantes, Nantes, France; 3 INSERM, IAME, UMR 1137, Paris, France; 4 Assistance Publique–Hôpitaux de Paris, Hôpital Bichat–Claude Bernard, Infectious Diseases Unit, Paris, Paris, France; 5 Equipe Operationnelle d'Hygiène, Siège Assistance Publique–Hôpitaux de Paris, Paris, France; 6 Universitaire Paris Diderot, Sorbonne Paris Cité, Paris, France; 7 Central Infection Control Team, Assistance Publique–Hôpitaux de Paris, Paris, France; 8 Equipe Mobile d’Infectiologie, Hôpital Cochin, Assistance Publique–Hôpitaux de Paris, Paris, France; 9 Equipe de Prévention du Risque Infectieux, Hôpital Bichat, Assistance Publique–Hôpitaux de Paris, Paris, France; 10 Assistance Publique–Hôpitaux de Paris, Hôpital Bichat–Claude Bernard, Infection Control Unit, Paris, France

## Abstract

**Importance:**

Controversy remains regarding the transmission routes of severe acute respiratory syndrome coronavirus 2 (SARS-CoV-2).

**Objective:**

To review current evidence on air contamination with SARS-CoV-2 in hospital settings and the factors associated with contamination, including viral load and particle size.

**Evidence Review:**

The MEDLINE, Embase, and Web of Science databases were systematically queried for original English-language articles detailing SARS-CoV-2 air contamination in hospital settings between January 1 and October 27, 2020. This study was conducted in accordance with the Preferred Reporting Items for Systematic Reviews and Meta-Analyses extension for Scoping Reviews (PRISMA-ScR) guidelines. The positivity rate of SARS-CoV-2 viral RNA and culture were described and compared according to the setting, clinical context, air ventilation system, and distance from patients. The SARS-CoV-2 RNA concentrations in copies per meter cubed of air were pooled, and their distribution was described by hospital areas. Particle sizes and SARS-CoV-2 RNA concentrations in copies or median tissue culture infectious dose (TCID50) per meter cubed were analyzed after categorization as less than 1 μm, from 1 to 4 μm, and greater than 4 μm.

**Findings:**

Among 2284 records identified, 24 cross-sectional observational studies were included in the review. Overall, 82 of 471 air samples (17.4%) from close patient environments were positive for SARS-CoV-2 RNA, with a significantly higher positivity rate in intensive care unit settings (intensive care unit, 27 of 107 [25.2%] vs non–intensive care unit, 39 of 364 [10.7%]; *P* < .001). There was no difference according to the distance from patients (≤1 m, 3 of 118 [2.5%] vs >1-5 m, 13 of 236 [5.5%]; *P* = .22). The positivity rate was 5 of 21 air samples (23.8%) in toilets, 20 of 242 (8.3%) in clinical areas, 15 of 122 (12.3%) in staff areas, and 14 of 42 (33.3%) in public areas. A total of 81 viral cultures were performed across 5 studies, and 7 (8.6%) from 2 studies were positive, all from close patient environments. The median (interquartile range) SARS-CoV-2 RNA concentrations varied from 1.0 × 10^3^ copies/m^3^ (0.4 × 10^3^ to 3.1 × 10^3^ copies/m^3^) in clinical areas to 9.7 × 10^3^ copies/m^3^ (5.1 × 10^3^ to 14.3 × 10^3^ copies/m^3^) in the air of toilets or bathrooms. Protective equipment removal and patient rooms had high concentrations per titer of SARS-CoV-2 (varying from 0.9 × 10^3^ to 40 × 10^3^ copies/m^3^ and 3.8 × 10^3^ to 7.2 × 10^3^ TCID50/m^3^), with aerosol size distributions that showed peaks in the region of particle size less than 1 μm; staff offices had peaks in the region of particle size greater than 4 μm.

**Conclusions and Relevance:**

In this systematic review, the air close to and distant from patients with coronavirus disease 2019 was frequently contaminated with SARS-CoV-2 RNA; however, few of these samples contained viable viruses. High viral loads found in toilets and bathrooms, staff areas, and public hallways suggest that these areas should be carefully considered.

## Key Points

**Question:**

What is the level of air contamination from severe acute respiratory syndrome coronavirus 2 (SARS-CoV-2) in different hospital areas, and what factors are associated with contamination?

**Findings:**

In this systematic review of 24 studies, 17% of air sampled from close patient environments was positive for SARS-CoV-2 RNA, with viability of the virus found in 9% of cultures.

**Meaning:**

In this study, air both close to and distant from patients with coronavirus disease 2019 was frequently contaminated with SARS-CoV-2 RNA; however, few of these samples contained viable viruses.

## Teaser

This systematic review distills the current evidence on air contamination with severe acute respiratory syndrome coronavirus 2 (SARS-CoV-2) in hospital settings and assesses the factors associated with contamination, including viral load and particle size.

## Introduction

The transmission modes of severe acute respiratory syndrome coronavirus 2 (SARS-CoV-2) remain controversial.^1^ At the emerging stage of the pandemic, many countries implemented high-level precautions, including airborne and contact precautions, to prevent the spread from patients to health care professionals (HCPs).^2^ An emerging understanding of SARS-CoV-2 epidemiology, which is primarily transmitted from person to person through droplets, led to recommendations for droplet precautions to care for patients hospitalized with coronavirus disease 2019 (COVID-19).^3^ However, separating transmission dynamics into the dichotomy of droplet vs airborne transmission is probably simplistic. In some circumstances, aerosol particles (<5 μm in diameter) may be produced by individuals with infection and travel more than the 1.50 m commonly used to define transmission routes and contaminate surfaces further away.^4^

Environmental airflow may ease the spread of large particles.^5^ The switch from airborne to droplet precautions, combined with a global shortage of face masks and respirators, fed the controversy regarding respiratory protections to prevent transmission of SARS-CoV-2.^6,7^ This generated a mistrust in personal protective equipment (PPE), particularly regarding surgical masks and their ability to protect HCPs from SARS-CoV-2 transmission. As the World Health Organization recently acknowledged, airborne transmission could occur in crowded and closed environments in the community. This raises the question of whether similar transmission could occur in the hospital.^1^ Viral contamination of the air surrounding patients with COVID-19 and HCPs in hospitals may have serious implications for outbreak control strategies. We reviewed the current evidence on air contamination with SARS-CoV-2 in hospital settings, the viral load, and associated factors to better assess the risk of cross-transmission of COVID-19 among HCPs and patients.

## Methods

### Search Strategy

We performed a systematic search of MEDLINE via PubMed, Embase, and Web of Science on October 27, 2020, with terms covering COVID-19 and air contamination in hospital settings in articles published between January 1 and October 27, 2020 (eAppendix in the Supplement). Because of potential delays in indexing of databases, we also searched selected infectious disease journals (eAppendix in the Supplement). We also searched some preprint servers, including BioRxiv and MedRxiv as well as the reference lists of identified articles to find reports of additional studies. We conducted this scoping systematic review in accordance with the Preferred Reporting Items for Systematic Reviews and Meta-analyses (PRISMA) extension for scoping reviews (eTable in the Supplement).

### Inclusion and Exclusion Criteria

We included all literature related to COVID-19 published in English between January 1, 2020, and October 27, 2020, without restrictions, including original articles, research letters, and comments. We excluded experimental methods and studies performed in dental and primary care settings.

### Article Selection and Data Extraction

Two reviewers (G.B. and N.P.S.) screened all titles, abstracts, and full-text articles independently and resolved disagreements by consensus or consultation with a third reviewer (J.C.L.). The following information was then extracted: (1) setting, (2) clinical context, (3) ventilation system, (4) number of air samples performed, (5) sampling method, (6) location of sampler and distance from patients, (7) duration and air volume sampled, (8) method of SARS-CoV-2 search, (9) positivity rate, (10) viral load (SARS-CoV-2 RNA copies per m^3^), and (11) viral culture results.

### Statistical Analysis

We conducted a descriptive analysis of the characteristics of the included literature. We described the setting, patient clinical contexts, ventilation, air sampling and SARS-CoV-2 search methods, and the qualitative and quantitative results according to settings and the hospital area. We categorized the location of air sampling in 5 classes of hospital areas: close patient environments (ie, patient rooms or bays), toilet or bathroom, clinical areas (ie, workstations, anterooms or buffer rooms, corridors, and other spaces in the clinical unit), staff areas (ie, changing rooms, staff rooms including office, meeting rooms, dining rooms, and other staff areas), public areas (hallways and other indoor and outdoor public areas). When possible, we also classified the setting as intensive care unit (ICU) vs non-ICU; the clinical context as severe or critical vs mild, moderate, or asymptomatic; the ventilation system as negative pressure vs natural or mechanical; and the distance from patients as 1 m or less vs greater than 1 to 5 m. The positivity rate of viral RNA and the viral culture were pooled, described, and compared according to categories using a χ^2^ test. The results of SARS-CoV-2 RNA concentrations in copies per meter cubed of air were pooled, and their distribution was described by hospital areas. The Kruskall-Wallis test was used to compare the nonnormally distributed RNA concentrations across hospital areas. A 2-tailed *P* < .05 was considered statistically significant. Studies presenting the combined results of particle sizes and SARS-CoV-2 RNA concentrations in copies or median tissue culture infectious dose (TCID50) per meter cubed were analyzed after categorization of sizes as less than 1 μm, 1 to 4 μm, and greater than 4 μm, the thresholds available across the 3 studies.

## Results

### Search Results

**Figure 1.**
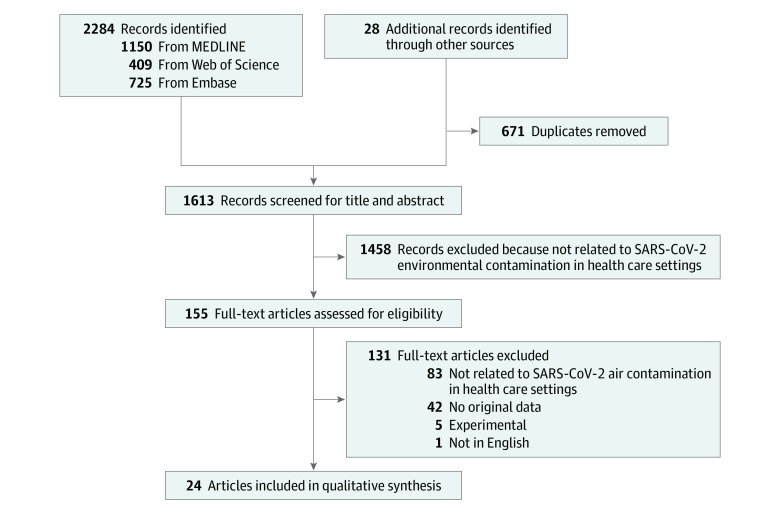
Flowchart of the Search Strategy

We identified 2284 records, 671 (29.4%) of which were excluded as duplicates. Title and abstract screening were conducted for the remaining 1613 articles, 1458 (90.4%) of which were excluded because they were unrelated to air contamination by SARS-CoV-2 in hospital settings. We retrieved the full text of the 155 remaining articles. After further screening and supplementary searching of articles published or posted between January 1 and October 27, 2020, we identified an additional article, and a total of 24 articles were included in the review (Figure 1).^3,8,9,10,11,12,13,14,15,16,17,18,19,20,21,22,23,24,25,26,27,28,29,30^

### Characteristics of Included Articles and Studies

Of the 24 included studies, all were cross-sectional observational studies. Ten studies (41.7%) were from China,^9,10,11,15,18,19,20,22,25,29^ and the remaining were from the United States (4 [16.7%]),^12,13,26,30^ Hong Kong (2 [8.3%]),^14,21^ Korea (2 [8.3%]),^24,27^ Singapore (2 [8.3%]),^3,16^ Iran (2 [8.3%]),^8,28^ the United Kingdom (1 [4.2%]),^19^ and Italy (1 [4.2%]).^23^ Of all included articles and studies, 20 (83.3%)^3,8,9,10,11,14,15,16,17,20,21,22,23,24,25,26,27,28,29,30^ were published in peer-reviewed journals, and 4 (16.7%)^12,13,17,18^ were posted on preprint servers.

A total of 23 studies (95.8%)^3,8,9,10,11,12,13,14,15,16,17,18,19,21,22,23,24,25,26,27,28,29,30^ sampled the air in the close patient environments, 12 (50.0%)^3,9,10,11,12,17,18,19,20,23,24,28^ in clinical areas away from patients, 8 (33.3%)^9,10,11,12,13,19,23,29^ in staff areas, 6 (25.0%)^3,9,17,18,19,22^ in toilets and/or bathrooms, and 6 (33.3%)^9,10,12,17,20,28^ in public areas (Table 1). The clinical context of patients hospitalized in the targeted areas was detailed in 18 studies, of which 10 (50.0%)^8,9,10,11,22,23,25,27,28,29^ were performed in units hospitalizing patients with severe or critical illness, 11 (61.1%)^3,11,12,14,15,16,24,25,26,28,30^ with patients with mild, moderate, or asymptomatic disease, and 4 studies (22.2%)^11,14,25,28^ with both categories.

**Table 1.** Summary of Included Studies Evaluating the Air Contamination With SARS-CoV-2 in the Hospital Environment

Source	Setting	Clinical context	Location	Air ventilation	Distance from patients, m	Duration (air volume per sample, L)	Microbiology	SARS-CoV-2		Positivity, No./total No. (%)
								Viral load, RNA copies/m^3^	Viral culture, No./total No. (%)	
**ICU patient environments**										
Liu et al,^9^ 2020	ICU	Severe	IR	Negative pressure	ND	5 h-7 d (1500-50 400 )	ddPCR	31 113	NA	2/3 (66)
Faridi et al,^8^ 2020	ICU	Severe	Multiple-bed room	Mechanical or natural	2-5	60 min (90)	RT-PCR; Ct, 38	NA	NA	0/9
Guo et al,^11^ 2020	ICU	Severe	IRs, bay room	12 Air supplies; 16 air discharges/h	ND	30 min (300)	RT-qPCR	Mean: 3.8 near air outlet; 1.4 near patients	NA	13/32 (41); Ct, 35.7 and 44.4
Lei et al,^22^ 2020	ICU	Critical	Multiple-bed room	ND	1	4 h (1260)	RT-qPCR	NA	NA	0/1; Ct, 41.5
Li et al,^10^ 2020	ICU	Severe	Multiple-bed room; 3 beds	12 Air supplies; 16 air discharges/h	1-5	5 h (21 600)	RT-PCR; Ct, <37 and >40	NA	NA	0/9
Zhou et al,^19^ 2020	ICU	ND	ND	Natural ventilation	1-5	40 min (600-16 000)	RT-qPCR; Ct, 39.5	NP	NA	0/5
Razzini et al,^23^ 2020	ICU	2 Patients intubated, 1 patient not intubated	Multiple-bed room	Negative pressure	ND	40 min (2000)	RT-PCR	Mean, 22.7	NA	12/12 (100)
Ding et al,^18^ 2020	ICU	Mild	IRs	Negative pressure	0.5	20-30 min (300-10 000)	RT-PCR	NA	NA	0/26
Ahn et al,^27^ 2020	ICU	3 Patients intubated	IRs	Negative pressure and 12 air changes/h	1	20 min (250)	rRT-PCR; Ct, <35; viral culture	NA	NP	0/3
Kenarhoohi et al,^28^ 2020	ICU	6 Critical	Multiple beds; 10 patients	Natural	≥2	180 min (2160)	RT-PCR; Ct, <40	NA	NA	0/6
Jin et al,^29^ 2020	ICU	1 Critical	IR	Negative pressure	0.5	15 min (6000)	qRT-PCR; Ct, <40	NA	NA	1/1 (100)
Tan et al,^25^ 2020	ICU	9 Severe or critical	Single room	Natural ventilation	1	60 min (300)	RT-PCR	NA	NA	1/10 (10)
**Non-ICU patient environments**										
Liu et al,^9^ 2020	GW	Severe	4 Single and 1 multiple-bed room; 2 beds	Negative pressure	ND	5 h (1500)	ddPCR	0	NA	0/2
Faridi et al,^8^ 2020	GW	Severe	Multiple-bed room; 2-9 beds	Mechanical or natural	2-5	60 min (90)	RT-PCR; Ct, 38	NA	NA	0/1
Ong et al,^3^ 2020	GW	Moderate or mild	IRs	12 Air exchanges/h	<1 and 2-5	4 h (3600)	RT-PCR; Ct, 45	ND	NA	0/18
Santarpia et al,^12^ 2020	IW	Mild	IRs	Negative pressure	Bedside table or desk	15 min (750)	RT-PCR; Ct, 45; viral culture	NA	0/10	0/18
Guo et al,^11^ 2020	GW	Mild	Single bay room	8 Air supplies and 12 air discharges/h	Near patients, near air outlet	30 min (300)	RT-qPCR	0.68 near air outlet	NA	2/16 (12.5)
Chia et al,^16^ 2020	GW	Mild or asymptomatic	IRs	12 Air changes/h	1 and 2.1	4 h (840)	RT-PCR	Mean (range), 1.3 (0.916-2)	NA	4/10 (40)
Lei et al,^22^ 2020	IW	Critical	ND	ND	1	4 h (1260)	RT-qPCR	NA	NA	1/1 (100); Ct, 44.6
Cheng et al,^14^ 2020	IW	Severe, mild, asymptomatic	IRs	12 Air changes/h, shelter	0.1	20 min (1000)	RT-PCR	0	NA	0/6
Cheng et al,^21^ 2020	IW	ND	IRs	Negative pressure	0.1	5 min (1000)	RT-PCR	0	NA	0/8
Zhou et al,^17^ 2020	IW and GW	ND	ND	ND	ND	ND (1000)	RT-qPCR; Ct, <40.4; viral culture	mean (range), 1.17 (0.16-7.04)	0/12	6/12 (50)
Li et al,^10^ 2020	IW	Severe	Multiple-bed room; 3 beds	8 Air supplies; 12 air discharges/h	1-5	5 h (21 600)	RT-PCR; Ct, <37 and >40	NA	NA	0/18
Wei et al,^15^ 2020	IW	Mild or asymptomatic	IRs	12 Air exchanges/h	0.6	15 min (1500)	RT-PCR; Ct, <35	NA	NA	0/6
Zhou et al,^19^ 2020	GW	ND	IRs	Natural	1-5	40 min (600-16 000)	RT-PCR; Ct, <39.5	NA	NA	0/16
Santarpia et al,^13^ 2020	ND	ND	IRs	ND	ND	30 min (ND)	RT-qPCR; viral culture	2.41 TCID50/cm^3^ of air	3/18 (17)	18/18 (100)
Kim et al,^24^ 2020	IW and GW	7 Mild and 1 asymptomatic	IRs; 5-bed room	IR with 15 air changes/h; IR without negative pressure; room without negative air pressure	2	20 min (1000)	rRT-PCR; Ct, <35	NA	NA	0/32
Tan et al,^25^ 2020	IW	15 Mild	3 Patients per room	Natural ventilation	1	60 min (300)	RT-PCR	NA	NA	1/2 (50)
Binder et al,^26^ 2020	IW	4 Asymptomatic; 16 mild	Single rooms	14 Air exchange/h	1, 1.4, 2.2, and 3.2	240 min (840)	RT-PCR; viral culture	NA	0/3	3/160 (1.9)
Kenarhoohi et al,^28^ 2020	Laboratory, radiology, internal medicine, emergency	5 Mild and 1 suspected case	Multiple rooms, 18-30 patients	Natural	≥2	180 min (2160)	RT-PCR; Ct, <40	NA	NA	0/6
Lednicky et al,^30^ 2020	IW	2 Patients with mild disease	IRs	6 Air changes/h	2-4.8	180 min (ND)	RT-PCR; viral culture	Mean (range), 46 (16-94); 2-74 TCID50 U/L of air	4/4 (100)	4/4 (100)
**Toilet or bathroom**										
Liu et al,^9^ 2020	Non-ICU	NA	Patient mobile toilet room	No ventilation	NA	20 h (6000)	ddPCR	1	NA	1/1 (100)
Ong et al,^3^ 2020	GW	NA	IRs	ND	NA	8 h (2400)	RT-PCR; Ct, 45	NA	NA	0/6
Lei et al,^22^ 2020	IW	NA	Patient bathroom	ND	NA	4 h (1680)	RT-qPCR	NA	NA	2/2 (100); Ct, 35.6, 35.5
Zhou et al,^17^ 2020	Cohort ward	NA	Outside patient bay, in the ward	ND	NA	ND (1000)	RT-qPCR; Ct, <40.4; viral culture	0.464	0/2	1/2 (50)
Zhou et al,^19^ 2020	Fever clinic	NA	In the ward	Natural	NA	40 (16 000)	RT-PCR; Ct, <39.5	NA	NA	0/3
Ding et al,^18^ 2020	4 ICU	NA	Patient bathroom	4 With negative pressure	NA	20-30 min (420-10 000)	RT-PCR	NA	NA	1/7 (14)
**Clinical areas**										
Liu et al,^9^ 2020	Non-ICU	ND	Workstation	Natural	ND	320-1200 min (1600-6000)	ddPCR	0, 1, 1, 5, 9	NA	4/5 (80)
Ong et al,^3^ 2020	ICU and non-ICU	ND	Corridor, anteroom	NA	ND	15 min (3000); 480 min (2400)	RT-PCR; Ct, 45	NA	NA	0/12
Santarpia et al,^12^ 2020	ND	Mild	Floor adjacent to rooms	NA	>6 ft	15 min (750)	RT-PCR; Ct, 45; viral culture	2.58, 3.76	0/3	2/3 (66)
Guo et al,^11^ 2020	ICU, GW	1 Severe, ND	Near office, pharmacy, nurse station, corridor, and buffer room	ND	ND	30 min (27 000-72 000)	RT-qPCR	0.54	NA	1/34 (3)
Wu et al,^20^ 2020	1 ICU	Ward and various clinical rooms	ND	ND	ND	30 min (ND)	RT-PCR; Ct, 43	NA	NA	0/69
Zhou et al,^17^ 2020	ICU and non-ICU	ND	Nurse station, ward, ambulatory waiting room, Resus bay, CPAP unit	ND	ND	ND (1000)	RT-qPCR; Ct, <40.4; viral culture	0.404, 0.035, 1.922, 0.031	0/10	4/10 (40)
Li et al,^10^ 2020	ICU and non-ICU	Severe	Corridor, clinic, buffer room	1 With negative pressure	ND	250 min (21 600)	RT-PCR; Ct, <37 and >40	NA	NA	0/45
Zhou et al,^19^ 2020	ND	ND	Corridor and preroom	Natural	ND	40 min (16 000)	RT-PCR; Ct, <39.5	NA	NA	0/18
Razzini et al,^23^ 2020	ND	ND	Corridor	ND	ND	40 min (16 000)	RT-PCR	NA	NA	8/8 (100); Ct, 31.1
Ding et al,^18^ 2020	IW	3 Mild, ND for others	Ward, corridor, nurse station, and storage room	3 Negative pressure, ND for others	ND	20-30 (300-10 000)	RT-PCR	NA	NA	1/12 (8); Ct, 37.8
Kim et al,^24^ 2020	IW and GW	7 Mild and 1 asymptomatic	5 Anterooms, regardless of room type	ND	ND	20 min (1000)	RT-PCR; Ct, <35	NA	NA	0/20
Kenarhoohi et al,^28^ 2020	ICU entrance	NA	NA	Natural	NA	180 min (2160)	RT-PCR; Ct, <40	NA	NA	0/1
**Staff areas**										
Liu et al,^9^ 2020	ICU	NA	Changing, meeting, and dining rooms, warehouse	Natural and/or mechanical; small air purifier	NA	300-1200 min (1500-6000)	ddPCR	Mean, 13.8	NA	10/13 (77)
Santarpia et al,^12^ 2020	IW	NA	Personal air sample	NA	ND	NA	RT-PCR; Ct, 45	Mean, 20.037	NA	4/4 (100)
Guo et al,^11^ 2020	ICU and GW	NA	Dressing rooms	NA	ND	30 min (45 000-108 000)	RT-qPCR	NA	NA	0/36
Santarpia et al,^13^ 2020	ND	NA	Staff and changing rooms	NA	ND	ND (1000)	RT-qPCR; Ct, <40.4; viral culture	0.249	0/4	1/4 (25)
Li et al,^10^ 2020	ICU and IW	NA	Conference room and clean zone	2/3 With negative pressure	ND	270-540 min (21 600-43 200)	RT-PCR; Ct, <37 and >40	NA	NA	0/45
Zhou et al,^19^ 2020	ND	NA	Waste storage	Natural	ND	40 min (16 000)	RT-PCR; Ct, <39.5	NA	NA	0/2
Razzini et al,^23^ 2020	ND	NA	Changing and locker rooms	NA	ND	40 min (8000-14 000)	RT-PCR	ND	NA	0/17
Jin et al,^29^ 2020	ICU	NA	Changing room	Negative pressure	Middle	15 min (6000)	qRT-PCR; Ct, <40	NA	NA	0/1
**Public areas**										
Liu et al,^9^ 2020	NA	NA	Pharmacy, hall, office, store, and supermarket	Mechanical, natural, outdoor	NA	300-1000 min (1500-5000)	ddPCR	3, 7, 11, 3	NA	4/11 (36)
Santarpia et al,^12^ 2020	NA	NA	Hallway	ND	NA	ND (ND)	RT-PCR; Ct, 45; viral culture	0.979-8.688	0/12	8/12 (67)
Wu et al,^20^ 2020	NA	NA	Public area	ND	NA	30 min (ND)	RT-PCR; Ct, 43	NA	NA	0/6
Zhou et al,^17^ 2020	NA	NA	Main entrance, toilet entrance, and lift area	ND	NA	ND (1000)	RT-qPCR; Ct, <40.4; viral culture	1.574, 1.545	0/3	2/3 (67)
Li et al,^10^ 2020	NA	NA	Public area	ND	NA	270 min (21 600)	RT-PCR; Ct, <37 and >40	NA	NA	0/9
Kenarhoohi et al,^28^ 2020	Hospital entrance	NA	NA	Natural	NA	180 min (2160)	RT-PCR; Ct, <40	NA	NA	0/1

Abbreviations: CPAP, continuous positive airway pressure; Ct, cycle threshold; ddPCR, droplet-digital polymerase chain reaction; GW, general ward; ICU, intensive care unit; IR, isolation room; IW, isolation ward; NA, not applicable; ND, not detailed; NP, not performed; RT-PCR, reverse transcription–polymerase chain reaction; RT-qPCR, quantitative reverse transcription–polymerase chain reaction; SARS-CoV-2, severe acute respiratory syndrome coronavirus 2.

A median of 24 air samples were collected per study, varying from 2 to 160 samples. In close patient environments, a median of 10 air samples (range, 1-160) were performed, 2.5 (range, 1-7) in toilets and/or bathrooms, 11 (range, 1-69) in clinical areas, 9 (range, 1-45) in staff areas, and 10 (1-12) in public areas. Overall, 19 studies (79.2%)^3,8,9,10,11,12,13,14,15,16,17,19,22,23,25,26,28,30^ sampled the air from non-ICU patient rooms, and 12 (50.0%)^8,9,10,11,18,19,22,23,25,27,28,29^ in ICU rooms. Among the 19 studies^3,8,9,10,11,12,14,15,18,19,21,23,24,25,26,27,28,29,30^ with the available information, 360 samples were taken in patient rooms with negative pressure and 66 with natural or mechanical ventilation. When pooling the 19 studies^3,8,10,11,12,14,15,16,18,19,22,24,25,26,27,28,29,30^ detailing the distance from patient, a total of 118 samples were performed 1 m or less from patients and 236 from greater than 1 to 5 m.

All included studies used reverse transcription–polymerase chain reaction (RT-PCR) to identify SARS-CoV-2 RNA, with a quantification of RNA copies per meters cubed or per liter in 8 studies (33.3%). One study^9^ used a droplet digital RT-PCR method. The viral culture was planned in the methods of 6 studies (20.8%)^12,13,17,26,27,30^ but performed in 5 (12.5%) of them.^12,13,17,26,30^ The remaining did not perform viral culture due to negative RT-PCR results. Three studies (12.5%)^9,13,16^ assessed the particle size in parallel to RNA concentration or viral titer.

### RT-PCR and Culture Results by Hospital Areas

A total of 893 air samples were performed across the 24 studies reviewed, including 471 (52.7%) in close patient environments, 237 (26.5%) in clinical areas, 122 (13.7%) in staff areas, 42 (4.7%) in public areas, and 21 (2.4%) in toilets and/or bathrooms (Table 2). Overall, 82 of 471 air samples (17.4%) from close patient environments were positive for SARS-CoV-2 RNA. Among the 107 samples performed in ICU rooms, 27 (25.2%) were positive vs 39 of 364 (10.7%) in non-ICU rooms (*P* < .001). The air RNA positivity rate was 47 of 360 (13.1%) in rooms with negative pressure and 6 of 66 (9.1%) in rooms with natural or mechanical ventilation. In toilets and/or bathrooms, 5 of 21 samples (23.8%) samples were positive. In clinical areas, the overall positivity rate was 8.4% (20 of 237), varying from 0 of 64 in anterooms or buffer rooms to 6 of 22 (27.2%) at workstations (*P* < .001). In staff areas, 15 of 122 samples (12.3%) were positive, with 5 of 26 (19.2%) in staff meeting rooms vs 2 of 51 (3.9%) in changing rooms and 8 of 45 (17.8%) in other types of staff rooms (*P* = .06). Overall, 14 of 42 samples (33.3%) in public areas were positive, with 9 of 16 (56.3%) in hallways, 2 of 18 (11.1%) in other indoor areas, and 3 of 8 (37.5%) in outdoor public areas (*P* = .01). A total of 81 viral cultures were performed across 3 studies (47 samples [58.0%] from close patient environment, 2 [2.5%] in toilets/bathroom, 13 [16.0%] in clinical areas, 4 [4.9%] in staff areas, and 15 [18.5%] in public areas). Two studies^13,30^ described positive viral cultures, both from the close patient environment (3 of 39 [7.7%];^13^ and 4 of 4 [100%]^30^) in a non-ICU setting.

**Table 2.** Description of Reverse Transcription–Polymerase Chain Reaction and Culture Results Categorized by Hospital Areas

Area	SARS-CoV-2				
	Viral RNA			Viral culture	
	No./total No.	Positivity, %	*P* value	No./total No.	Positivity, %
Patient environments					
All	82/471	17.4	NA	7/47	14.9
Ward					
ICU	27/107	25.2	<.001	NA	NA
Non-ICU	39/364	10.7		7/47	14.9
Ventilation					
Negative pressure	47/360	13.1	.37	0/13	0
Mechanical or natural	6/66	9.1		4/4	100
Distance from patient, m					
≤1	3/118	2.5	.22	NA	NA
1-5	13/236	5.5		4/7	57.1
Clinical context					
Severe or critical	20/96	20.8	<.001	NA	NA
Mild, moderate, or asymptomatic	23/303	7.6		4/17	23.5
Patient toilets or bathrooms	5/21	23.8	NA	0/2	0
Clinical areas					
All	20/237	8.4	NA	0/13	0
Corridor	9/48	18.7	<.001	NA	NA
Workstation	6/22	27.2		0/5	0
Anteroom or buffer room	0/64	0		NA	NA
Others	5/103	4.8		0/8	0
Staff areas					
All	15/122	12.3	NA	0/4	0
Changing room	2/51	3.9	.06	0/1	0
Meeting or staff room	5/26	19.2		0/3	0
Others	8/45	17.8		NA	NA
Public areas					
All	14/42	33.3	NA	0/15	0
Hallways	9/16	56.2	.01	0/14	0
Other, indoor	2/18	11.1		0/1	0
Outdoor	3/8	37.5		NA	NA

Abbreviations: ICU, intensive care unit; NA, not applicable; SARS-CoV-2, severe acute respiratory syndrome coronavirus 2.

### SARS-CoV-2 RNA Concentrations in Copies per Meters Cubed of Air, According to Hospital Areas

**Figure 2.**
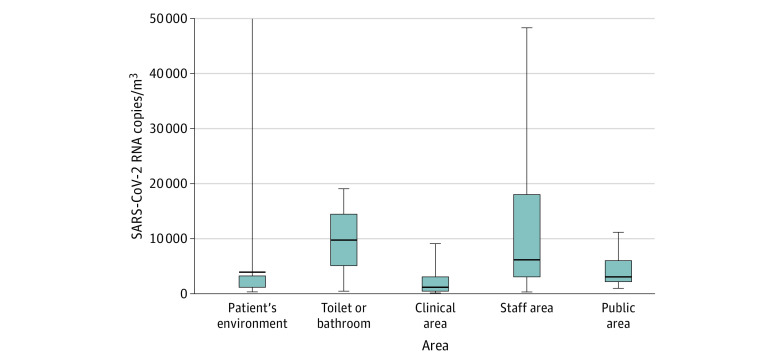
Distribution of Pooled Severe Acute Respiratory Syndrome Coronavirus 2 (SARS-CoV-2) RNA Concentrations in Copies per Meter Cubed of Air, by Hospital Area In patient’s environment, there was an outlier, with 1 sample finding 94 000 SARS-CoV-2 RNA copies/m^3^ in 1 non–intensive care unit room.^30^

**Figure 3.**
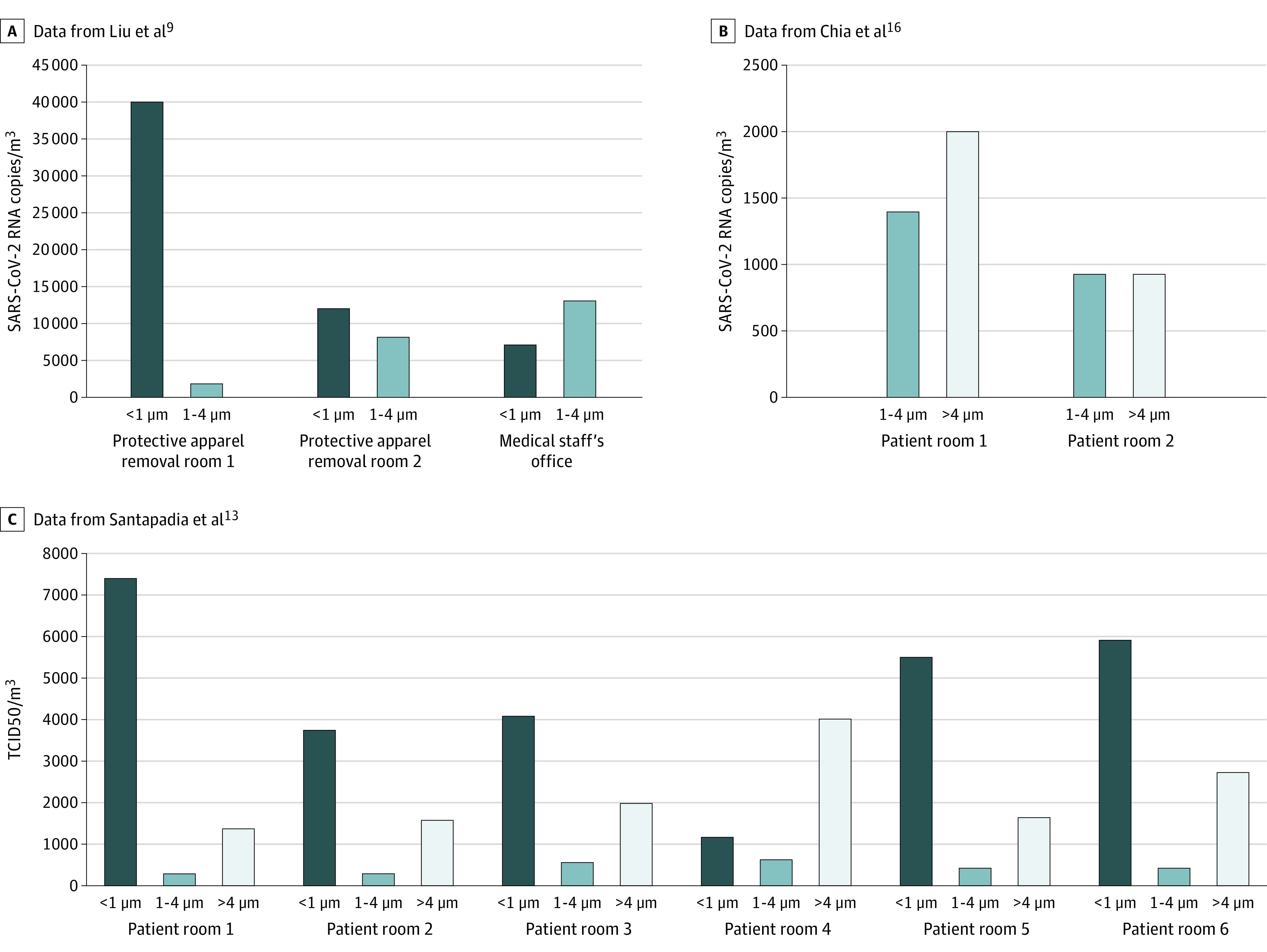
Concentration of Airborne Severe Acute Respiratory Syndrome Coronavirus 2 (SARS-CoV-2) in Different Aerosol Sizes TCID50 indicates median tissue culture infectious dose.

Among studies with SARS-CoV-2 positive air samples^11,12,13,16,17,23,30^ that performed a quantitative RT-PCR, the median (interquartile range [IQR]) RNA concentrations varied from 1.0 × 10^3^ copies/m^3^ (0.4 × 10^3^ to 3.1 × 10^3^) in clinical areas to 9.7 × 10^3^ (5.1 × 10^3^ to 14.3 × 10^3^) in the air of toilets and/or bathrooms (Figure 2). The median (IQR) concentration found in close patients environments was 3.8 × 10^3^ (1.2 × 10^3^ to 3.3 × 10^3^) copies/m^3^ (*P* < .001). Among the 3 studies^9,13,16^ that assessed the particle size in air sampled in parallel with the viral load, 1 study^16^ found an RNA concentration of 2.0 × 10^3^ copies/m^3^ for particles greater than 4 μm and 1.3 × 10^3^ for particles sized 1 to 4 μm in 1 patient room, and 927 and 916 copies/m^3^ of those sizes, respectively, in a second room, both at a distance of 1.0 to 2.1 m from patients (Figure 3). A second study^9^ of 2 PPE removal rooms found 40.0 × 10^3^ and 12.0 × 10^3^ copies/m^3^ for particles less than 1 μm, and 2.0 × 10^3^ to 8.0 × 10^3^ copies/m^3^ for particles sized 1 to 4 μm in 2 PPE removal rooms. A concentration of 7.0 × 10^3^ copies/m^3^ was found for particles less than 1 μm and 13.0 × 10^3^ copies/m^3^ for particles sized 1 to 4 μm in medical staff offices.^31^ For the third study that performed viral cultures with air samples from 6 different patients’ room,^13^ the median (IQR) viral concentration was 4.8 (3.3-5.8) TCID50/m^3^ for particles less than 1 μm, 4.27 (2.96-5.48) TCID50/m^3^ for particles sized 1 to 4 μm, and 1.82 (1.6-2.55) TCID50/m^3^ for particles greater than 4 μm.^13^

## Discussion

This scoping systematic review of the literature suggests that air near and distant from patient environments, including toilets and/or bathrooms, staff areas, and public areas, may carry viral RNA. However, the infectivity of the virus assessed by viral culture was only reported by 2 studies in non-ICU patient rooms. PPE removal and patient rooms had high concentrations per titer of SARS-CoV-2 with aerosol size distributions that showed peaks of particles sized less than 1 μm; for staff offices, the size distribution peaked for particles sized greater than 4 μm.

The results of positivity rate in ICU and non-ICU patient environments were highly heterogeneous and appeared superior in the ICU when pooling the results. In the ICU, 7 of 12 studies did not find SARS-CoV-2 RNA, whereas the remaining did, with 37.5% to 100% positive samples. In non-ICU patient environments, 11 of 19 did not find SARS-CoV-2 RNA, and 8 studies found viral RNA present in from 1.9% to 100% of samples. This heterogeneity may be explained either by a different case mix or by a difference in the methods used for air sampling. The level of severity of patients’ infections was not associated with increased air contamination. Several studies^32,33^ suggested higher viral loads might be associated with severe clinical outcomes. However, the association between clinical conditions and air contamination may be more complex. The potential opportunistic airborne contamination occurring during aerosol-generating procedures (AGPs) and ventilation at the time of sampling could inform the results. All these factors were poorly detailed in the articles analyzed. The sampling method, including the sampler used; its position in the clinical unit and in relation to patients; the duration of sampling; the volume sampled; and the conditions for transfer to the laboratory were highly variable across studies. The volume of a single room is approximately 40 m^3^. However, most sample volumes were less than 10 m^3^, at various airflow rates, for a duration of less than or equal to 1 hour, potentially not reflecting the reality of air contamination. The climatic conditions (eg, temperature and hygrometry) were poorly detailed in studies reviewed, but they may affect the capacity for viral particles to persist in the air.^34^ The methods for RNA detection varied, especially the cycle threshold (Ct) for PCR positivity, which also varied from 37 to 45. The RT-PCR Ct values are strongly associated with a cultivable virus. The probability of culturing virus declines to 8% in samples with Ct of greater than 35.^35^ Only 2 studies^13,30^ described a positive viral culture on samples with SARS-CoV-2 RNA on RT-PCR, suggesting that most samples did not contain enough infectious virus. Most sampling methods affect viral infectivity, which may partly explain these results.^36^ Future studies should consider these points for better accuracy and comparability of data.

The concentration of SARS-CoV-2 RNA in aerosols detected in isolation wards and in areas where patients were receiving ventilation was very low. However, a higher concentration of viral RNA was found in patient toilets, public areas, and in some medical staff areas. The finding of high concentrations in staff rooms (ie, meeting and dining rooms) is consistent with the possible cross-transmission of COVID-19 among HCPs during breaks. During these periods, face masks are frequently removed in small areas without ventilation. Toilets and staff rooms are often small and poorly ventilated. The presence of SARS-CoV-2 RNA in stool samples has been described in several studies.^37,38^ Toilet flushing may lead to the aerosolization of RNA in small and nonventilated toilets or bathrooms. In an epidemic setting, public areas are often crowded, with both a high patient flow and high incidence of COVID-19. These factors have to be considered to control the transmission of COVID-19 between nonmasked HCPs in hospitals, especially staff rooms and lockers.

Only 3 studies^9,13,16^ assessed the size of particles found when searching for SARS-CoV-2. Regarding aerosols of submicrometer size that were observed in PPE removal and patient rooms, the authors of those studies hypothesized the resuspension of virus-laden aerosols from the surfaces of PPE worn by medical staff. The submicrometer virus–laden aerosols may originally come from direct deposition of respiratory droplets or airborne SARS-CoV-2 from a patient to the PPE. On the other hand, floor-deposited SARS-CoV-2 could be the source of virus-laden aerosols greater than 4 μm that were then carried across different areas by medical staff.

The findings of this scoping systematic review are consistent with the accumulated knowledge on other respiratory viruses. SARS-CoV-1 is commonly recognized to be mainly transmitted through large droplets, requiring particular conditions to be airborne transmitted, such as AGPs.^39,40^ For other respiratory viruses, a 2019 review described the frequent presence of nucleic material (RNA or DNA) in the air around patients with influenza, respiratory syncytial virus, adenovirus, rhinovirus, and other coronaviruses but rarely the presence of viable viruses.^41^ The current available evidence on hospital air contamination by SARS-CoV-2 leans toward the effectiveness of surgical face masks in most circumstances to prevent cross-transmission of COVID-19 in hospital settings.^42^ In contrast, AGPs on the respiratory tract require wearing a respirator (N95 or FFP2) to prevent transmission and protect HCPs.^5,43^ However, the validation of these hypothesis regarding the transmission mode of COVID-19 and the associated efficacy of PPE requires more robust studies. A randomized clinical trial comparing the surgical face mask with respirator may provide important information for recommendations regarding respiratory protection for HCPs in settings in addition to AGPs. Assessing SARS-CoV-2 RNA and viable virus contamination of surgical face masks and respirators worn by HCP according to a panel of procedures with patients with COVID-19 would provide information on exposure in routine practice.

### Limitations

This study has limitations. First, the context (ie, location, ventilation, distance, and clinical context) were infrequently detailed in studies. Misclassification may have occurred when variables were categorized without enough detail. Moreover, the sampling and microbiology methods were highly heterogeneous across studies. As explained earlier, these flaws potentially affected the comparability of data and the reliability of pooled data analysis. This issue was avoided by performing categorization only when data were available. Second, for a better clarity of analysis, we did not include surface contamination. However, air and surface contamination are potentially correlated and may ease the understanding of resuspension. Third, we included articles not validated by a peer review process.

## Conclusions

In this study, the air around patients hospitalized with COVID-19 was frequently contaminated with SARS-CoV-2 RNA but rarely with viable viruses. The available data suggest that COVID-19 requires particular conditions to be transmitted through the air (such as AGPs), leaning toward the effectiveness of surgical face masks in most circumstances. High viral loads found in toilets and/or bathrooms, staff areas, and public hallways argue for a careful consideration of these areas for the prevention of COVID-19 transmission. However, the presence of viable viruses should be primarily considered, given that it is a required link for the potential of cross-transmission.

## Notes

Supplement. **eAppendix.** Supplementary Methods **eTable.** Evaluation of the Quality of Included Studies

## References

[1] World Health Organisation Transmission of SARS-CoV-2: implications for infection prevention precautions. Published online July 9, 2020. Accessed November 23, 2020. https://www.who.int/news-room/commentaries/detail/transmission-of-sars-cov-2-implications-for-infection-prevention-precautions

[2] Birgand G, Mutters NT, Otter JA, Analysis of national and international guidelines on respiratory protection equipment for COVID-19 in healthcare settings. medRxiv. Preprint published online April 29, 2020. doi:10.1101/2020.04.23.20077230

[3] Ong SWX, Tan YK, Chia PY, Air, surface environmental, and personal protective equipment contamination by severe acute respiratory syndrome coronavirus 2 (SARS-CoV-2) from a symptomatic patient. JAMA. 2020;323(16):1610-1612. doi:10.1001/jama.2020.3227 32129805 PMC7057172

[4] Bourouiba L Turbulent gas clouds and respiratory pathogen emissions: potential implications for reducing transmission of COVID-19. JAMA. 2020;323(18):1837-1838. doi:10.1001/jama.2020.4756 32215590

[5] Seto WH Airborne transmission and precautions: facts and myths. J Hosp Infect. 2015;89(4):225-228. doi:10.1016/j.jhin.2014.11.005 25578684 PMC7132528

[6] Morawska L, Tang JW, Bahnfleth W, How can airborne transmission of COVID-19 indoors be minimised? Environ Int. 2020;142:105832. doi:10.1016/j.envint.2020.105832 32521345 PMC7250761

[7] Chagla Z, Hota S, Khan S, Mertz D; International Hospital and Community Epidemiology Group Airborne transmission of COVID-19. Clin Infect Dis. 2020;ciaa1118. doi:10.1093/cid/ciaa1118 32780799 PMC7454327

[8] Faridi S, Niazi S, Sadeghi K, A field indoor air measurement of SARS-CoV-2 in the patient rooms of the largest hospital in Iran. Sci Total Environ. 2020;725:138401. doi:10.1016/j.scitotenv.2020.138401 32283308 PMC7194859

[9] Liu Y, Ning Z, Chen Y, Aerodynamic analysis of SARS-CoV-2 in two Wuhan hospitals. Nature. 2020;582(7813):557-560. doi:10.1038/s41586-020-2271-3 32340022

[10] Li YH, Fan YZ, Jiang L, Wang HB Aerosol and environmental surface monitoring for SARS-CoV-2 RNA in a designated hospital for severe COVID-19 patients. Epidemiol Infect. 2020;148:e154. doi:10.1017/S0950268820001570 32660668 PMC7371847

[11] Guo Z-D, Wang Z-Y, Zhang S-F, Aerosol and surface distribution of severe acute respiratory syndrome coronavirus 2 in hospital wards, Wuhan, China, 2020. Emerg Infect Dis. 2020;26(7):1583-1591. doi:10.3201/eid2607.200885 32275497 PMC7323510

[12] Santarpia JL, Rivera DN, Herrera V, Aerosol and surface transmission potential of SARS-CoV-2. medRxiv. Preprint published online June 3, 2020. doi:10.1101/2020.03.23.20039446

[13] Santarpia JL, Herrera VL, Rivera DN, The infectious nature of patient-generated SARS-CoV-2 aerosol. medRxiv. Preprint published online July 21, 2020. doi:10.1101/2020.07.13.20041632

[14] Cheng VC-C, Wong S-C, Chan VW-M, Air and environmental sampling for SARS-CoV-2 around hospitalized patients with coronavirus disease 2019 (COVID-19). Infect Control Hosp Epidemiol. 2020;41(11):1258-1265. doi:10.1017/ice.2020.282 32507114 PMC7327164

[15] Wei L, Lin J, Duan X, Asymptomatic COVID-19 patients can contaminate their surroundings: an environment sampling study. mSphere. 2020;5(3):e00442-20. doi:10.1128/mSphere.00442-20 32581071 PMC7316493

[16] Chia PY, Coleman KK, Tan YK, Detection of air and surface contamination by SARS-CoV-2 in hospital rooms of infected patients. Nature Communications. 2020;11:2800. doi:10.1038/s41467-020-16670-2 PMC7260225 32472043

[17] Zhou J, Otter JA, Price JR, Investigating SARS-CoV-2 surface and air contamination in an acute healthcare setting during the peak of the COVID-19 pandemic in London. Clin Infect Dis. 2020;ciaa905. doi:10.1093/cid/ciaa905 32634826 PMC7454437

[18] Ding Z, Qian H, Xu B, Toilets dominate environmental detection of SARS-CoV-2 virus in a hospital. medRxiv. Preprint published online April 7, 2020. doi:10.1101/2020.04.03.20052175

[19] Zhou L, Yao M, Zhang X, Detection of SARS-CoV-2 in exhaled breath from COVID-19 patients ready for hospital discharge. medRxiv. Preprint published online June 2, 2020. doi:10.1101/2020.05.31.20115196

[20] Wu S, Wang Y, Jin X, Tian J, Liu J, Mao Y Environmental contamination by SARS-CoV-2 in a designated hospital for coronavirus disease 2019. Am J Infect Control. 2020;48(8):910-914. doi:10.1016/j.ajic.2020.05.003 32407826 PMC7214329

[21] Cheng VCC, Wong S-C, Chen JHK, Escalating infection control response to the rapidly evolving epidemiology of the coronavirus disease 2019 (COVID-19) due to SARS-CoV-2 in Hong Kong. Infect Control Hosp Epidemiol. 2020;41(5):493-498. doi:10.1017/ice.2020.58 32131908 PMC7137535

[22] Lei H, Ye F, Liu X, SARS-CoV-2 environmental contamination associated with persistently infected COVID-19 patients. Influenza Other Respir Viruses. 2020;14(6):688-699. doi:10.1111/irv.12783 32578948 PMC7361718

[23] Razzini K, Castrica M, Menchetti L, SARS-CoV-2 RNA detection in the air and on surfaces in the COVID-19 ward of a hospital in Milan, Italy. Sci Total Environ. 2020;742:140540. doi:10.1016/j.scitotenv.2020.140540 32619843 PMC7319646

[24] Kim UJ, Lee SY, Lee JY, Air and environmental contamination caused by COVID-19 patients: a multi-center study. J Korean Med Sci. 2020;35(37):e332. doi:10.3346/jkms.2020.35.e332 32959546 PMC7505729

[25] Tan L, Ma B, Lai X, Air and surface contamination by SARS-CoV-2 virus in a tertiary hospital in Wuhan, China. Int J Infect Dis. 2020;99:3-7. doi:10.1016/j.ijid.2020.07.027 32730827 PMC7384415

[26] Binder RA, Alarja NA, Robie ER, Environmental and aerosolized severe acute respiratory syndrome coronavirus 2 among hospitalized coronavirus disease 2019 patients. J Infect Dis. 2020;222(11):1798-1806. doi:10.1093/infdis/jiaa575 32905595 PMC7499634

[27] Ahn JY, An S, Sohn Y, Environmental contamination in the isolation rooms of COVID-19 patients with severe pneumonia requiring mechanical ventilation or high-flow oxygen therapy. J Hosp Infect. 2020;106(3):570-576. doi:10.1016/j.jhin.2020.08.014 32828864 PMC7441047

[28] Kenarkoohi A, Noorimotlagh Z, Falahi S, Hospital indoor air quality monitoring for the detection of SARS-CoV-2 (COVID-19) virus. Sci Total Environ. 2020;748:141324. doi:10.1016/j.scitotenv.2020.141324 32805566 PMC7387923

[29] Jin T, Li J, Yang J, SARS-CoV-2 presented in the air of an intensive care unit (ICU). Sustain Cities Soc. 2020;102446. doi:10.1016/j.scs.2020.102446 32837871 PMC7428766

[30] Lednicky JA, Lauzardo M, Fan ZH, Viable SARS-CoV-2 in the air of a hospital room with COVID-19 patients. Int J Infect Dis. 2020;100:476-482. doi:10.1016/j.ijid.2020.09.025 32949774 PMC7493737

[31] Lui RN, Wong SH, Sánchez-Luna SA, Overview of guidance for endoscopy during the coronavirus disease 2019 pandemic. J Gastroenterol Hepatol. 2020;35(5):749-759. doi:10.1111/jgh.15053 32233034

[32] Liu Y, Yan L-M, Wan L, Viral dynamics in mild and severe cases of COVID-19. Lancet Infect Dis. 2020;20(6):656-657. doi:10.1016/S1473-3099(20)30232-2 32199493 PMC7158902

[33] Wölfel R, Corman VM, Guggemos W, Virological assessment of hospitalized patients with COVID-2019. Nature. 2020;581(7809):465-469. doi:10.1038/s41586-020-2196-x 32235945

[34] Tang S, Mao Y, Jones RM, Aerosol transmission of SARS-CoV-2? evidence, prevention and control. Environ Int. 2020;144:106039. doi:10.1016/j.envint.2020.106039 32822927 PMC7413047

[35] Singanayagam A, Patel M, Charlett A, Duration of infectiousness and correlation with RT-PCR cycle threshold values in cases of COVID-19, England, January to May 2020. Euro Surveill. 2020;25(32). doi:10.2807/1560-7917.ES.2020.25.32.2001483 32794447 PMC7427302

[36] Verreault D, Moineau S, Duchaine C Methods for sampling of airborne viruses. Microbiol Mol Biol Rev. 2008;72(3):413-444. doi:10.1128/MMBR.00002-08 18772283 PMC2546863

[37] Wang W, Xu Y, Gao R, Detection of SARS-CoV-2 in different types of clinical specimens. JAMA. 2020;323(18):1843-1844. doi:10.1001/jama.2020.3786 32159775 PMC7066521

[38] Wu Y, Guo C, Tang L, Prolonged presence of SARS-CoV-2 viral RNA in faecal samples. Lancet Gastroenterol Hepatol. 2020;5(5):434-435. doi:10.1016/S2468-1253(20)30083-2 32199469 PMC7158584

[39] Roy CJ, Milton DK Airborne transmission of communicable infection—the elusive pathway. N Engl J Med. 2004;350(17):1710-1712. doi:10.1056/NEJMp048051 15102996

[40] Booth TF, Kournikakis B, Bastien N, Detection of airborne severe acute respiratory syndrome (SARS) coronavirus and environmental contamination in SARS outbreak units. J Infect Dis. 2005;191(9):1472-1477. doi:10.1086/429634 15809906 PMC7202477

[41] Shiu EYC, Leung NHL, Cowling BJ Controversy around airborne versus droplet transmission of respiratory viruses: implication for infection prevention. Curr Opin Infect Dis. 2019;32(4):372-379. doi:10.1097/QCO.0000000000000563 31259864

[42] Temkin E; Healthcare Worker COVID-19 Surveillance Working Group Extremely low prevalence of asymptomatic COVID-19 among healthcare workers caring for COVID-19 patients in Israeli hospitals: a cross-sectional study. Clin Microbiol Infect. 2020;S1198-743X(20)30593-0. doi:10.1016/j.cmi.2020.09.040 33010442 PMC7527279

[43] Seto WH, Tsang D, Yung RWH, ; Advisors of Expert SARS group of Hospital Authority Effectiveness of precautions against droplets and contact in prevention of nosocomial transmission of severe acute respiratory syndrome (SARS). Lancet. 2003;361(9368):1519-1520. doi:10.1016/S0140-6736(03)13168-6 12737864 PMC7112437

