# Error in Figure

**DOI:** 10.1001/jamanetworkopen.2020.37904

**Published:** 2021-01-26

In the Original Investigation titled “Assessment of Air Contamination by SARS-CoV-2 in Hospital Settings,”^1^ published December 23, 2020, there was an error in Figure 3. In panel C, the y-axis markers should have read 1, 2, 3, 4, 5, 6, 7, and 8. This article has been corrected.^1^
