# Supplementary material for: Assessment of Air Contamination by SARS-CoV-2 in Hospital Settings
Source: JAMA Netw Open. 2020 Dec 23;3(12):e2033232. doi: 10.1001/jamanetworkopen.2020.33232 (PMC7758808; doi:10.1001/jamanetworkopen.2020.33232)
Supplement: Supplement. — eAppendix. Supplementary Methods eTable. Evaluation of the Quality of Included Studies [file jamanetwopen-e2033232-s001.pdf]

# Supplemental Online Content

Birgand G, Peiffer-Smadja N, Fournier S, Kerneis S, Lescure FX, Lucet JC. Assessment of air contamination by SARS-CoV-2 in hospital settings. *JAMA Netw Open*. 2020;3(12):e2033232. doi:10.1001/jamanetworkopen.2020.33232

**eAppendix.** Supplementary Methods
**eTable.** Evaluation of the Quality of Included Studies

This supplemental material has been provided by the authors to give readers additional information about their work.

© 2020 Birgand G et al. *JAMA Network Open.*

**eAppendix.** Supplementary Methods

**Medline search algorithm (n=1150)**

(“Severe Acute Respiratory Syndrome Coronavirus 2” OR “SARS-CoV-2” OR “COVID-19” OR ‘severe acute respiratory syndrome coronavirus 2’ [Supplementary Concept] OR ‘COVID-19’ [Supplementary Concept])
AND
(“Droplets” OR “Air” OR “Airborne” OR “Airborne Transmission” OR “Aerodynamic” OR “Aerosol” OR “airborne infection” OR “Environmental contamination” OR “Sampling” OR "Air Microbiology" [Mesh])
AND
(“health workers” OR “Hospital” OR “Hospital Rooms” OR “isolation rooms” OR “healthcare setting” OR "Hospitals"[Mesh] OR "Health Care Facilities, Manpower, and Services"[Mesh] OR “healthcare settings” OR “patients”)
AND (‘2019/12/01’[Date - Publication] : ‘2020/07/15’[Date - Publication])

**Web of science search algorithm (n=409)**

(TS=(Severe Acute Respiratory Syndrome Coronavirus 2) OR TS=(SARS-CoV-2) OR TS=(COVID-19))
AND
(TS=(Droplets) OR TS=(Air) OR TS=(Airborne) OR TS=(Airborne Transmission) OR TS=(Aerodynamic) OR TS=(Aerosol) OR TS=(airborne infection) OR TS=(Environmental contamination) OR TS=(Sampling))
AND
(TS=(health workers) OR TS=(Hospital) OR TS=(Hospital Rooms) OR TS=(isolation rooms) OR TS=(healthcare setting) OR TS=(patients))

**Embase search algorithm (n=725)**

('Severe Acute Respiratory Syndrome Coronavirus 2' OR 'SARS-CoV-2' OR 'COVID-19')
AND
('Droplets' OR 'Air' OR 'Airborne' OR 'Airborne Transmission' OR 'Aerodynamic' OR 'Aerosol' OR 'airborne infection' OR 'Environmental contamination' OR 'Sampling')
AND
('health workers' OR 'Hospital' OR 'Hospital Rooms' OR 'isolation rooms' OR 'healthcare setting' OR 'patients')

© 2020 Birgand G et al. *JAMA Network Open.*

**Infectious diseases journals searched**

| No | Journal | URL |
|---|---|---|
| **1** | LANCET INFECTIOUS DISEASES | https://www.thelancet.com/journals/laninf/home |
| **2** | CLINICAL INFECTIOUS DISEASES | https://academic.oup.com/cid |
| **3** | EUROSURVEILLANCE | https://www.eurosurveillance.org/ |
| **4** | EMERGING INFECTIOUS DISEASES | https://wwwnc.cdc.gov/eid/ |
| **5** | CLINICAL MICROBIOLOGY AND INFECTION | https://www.clinicalmicrobiologyandinfection.com/ |
| **6** | JOURNAL OF ANTIMICROBIAL CHEMOTHERAPY | https://academic.oup.com/jac |
| **7** | JOURNAL OF INFECTION | https://www.journalofinfection.com/ |
| **8** | JOURNAL OF INFECTIOUS DISEASES | https://academic.oup.com/jid |
| **9** | JOURNAL OF HOSPITAL INFECTION | https://www.journalofhospitalinfection.com/ |
| **10** | ANTIMICROBIAL RESISTANCE AND INFECTION CONTROL | https://aricjournal.biomedcentral.com/ |
| **11** | INFECTION CONTROL AND HOSPITAL EPIDEMIOLOGY | https://www.cambridge.org/core/journals/infection-control-and-hospital-epidemiology |
| **12** | AMERICAN JOURNAL OF INFECTION CONTROL | https://www.ajicjournal.org/ |

© 2020 Birgand G et al. *JAMA Network Open.*

**eTable. Evaluation of the Quality of Included Studies**

Use the rating scheme listed below with ratings of 1-5 for Reviews that include individual studies (modified from the Oxford Centre for Evidence-based Medicine for ratings of individual studies).

| Reference | First author | Study type | Quality Rating |
|---|---|---|---|
| 8 | Faridi | cross-sectional study | 4 |
| 9 | Liu | cross-sectional study | 4 |
| 10 | Li | cross-sectional study | 4 |
| 11 | Guo | cross-sectional study | 4 |
| 12 | Santarpia | cross-sectional study | 4 |
| 14 | Cheng | cross-sectional study | 4 |
| 3 | Ong | cross-sectional study | 4 |
| 15 | Wei | cross-sectional study | 4 |
| 16 | Chia | cross-sectional study | 4 |
| 19 | Zhou | cross-sectional study | 4 |
| 20 | Wu | cross-sectional study | 4 |
| 21 | Cheng | cross-sectional study | 4 |
| 17 | Zhou | cross-sectional study | 4 |
| 22 | Lei | cross-sectional study | 4 |
| 23 | Razzini | cross-sectional study | 4 |
| 13 | Santarpia | cross-sectional study | 4 |
| 18 | Ding | cross-sectional study | 4 |
| 24 | Kim | cross-sectional study | 4 |
| 25 | Tan | cross-sectional study | 4 |
| 26 | Binder | cross-sectional study | 4 |
| 27 | Ahn | cross-sectional study | 4 |
| 28 | Kenarkoohi | cross-sectional study | 4 |
| 29 | Jin | cross-sectional study | 4 |
| 30 | Lednicky | cross-sectional study | 4 |

© 2020 Birgand G et al. *JAMA Network Open.*
